# Nonalcoholic Fatty Liver Disease and Its Complex Relation with Type 2 Diabetes Mellitus—From Prevalence to Diagnostic Approach and Treatment Strategies

**DOI:** 10.3390/jcm11175144

**Published:** 2022-08-31

**Authors:** Cosmina-Theodora Diaconu, Cristian Guja

**Affiliations:** 1 Department of Diabetes, Nutrition and Metabolic Diseases, “Prof. Dr. N.C. Paulescu” National Institute of Diabetes, Nutrition and Metabolic Diseases, 030167 Bucharest, Romania; 2 Doctoral School of “Carol Davila” University of Medicine and Pharmacy, Dionisie Lupu 37, 020021 Bucharest, Romania; 3 Department of Diabetes, Nutrition and Metabolic Diseases, “Carol Davila” University of Medicine and Pharmacy, 020021 Bucharest, Romania

**Keywords:** Nonalcoholic Fatty Liver Disease (NAFLD), Type 2 Diabetes Mellitus (T2DM), Metabolic Syndrome-Associated Fatty Liver Disease (MAFLD), treatment

## Abstract

Prevalence of Nonalcoholic Fatty Liver Disease (NAFLD) and Type 2 Diabetes Mellitus (T2DM) are increasing rapidly worldwide, reaching epidemic proportions. Their association, based on common metabolic risk factors (obesity, insulin resistance (IR), unhealthy lifestyle), brings an additional risk of both hepatic and cardiovascular (CV) adverse clinical outcomes. The terminology of “NAFLD” is stigmatizing to some but not all patients, and a more practical one should be announced soon. Medical strategies can address both diseases simultaneously, as they have crossing pathophysiological mechanisms, mainly IR. Strategies vary from lifestyle intervention and pharmacological options, as more molecules designated for T2DM treatment may be helpful in NAFLD, to surgical procedures. This review focuses on the coexistence of NAFLD and T2DM, pointing out the utility of the appropriate terminology, its prevalence, and mortality rates among the diabetic population. Briefly, we have discussed the main pathophysiological mechanisms and the risk stratification algorithm for the development of NAFLD and nonalcoholic steatohepatitis (NASH) as well as the tools for evaluation of fibrosis. Finally, we have focused on the current therapeutic options for the treatment of NAFLD associated with T2DM.

## 1. Introduction

The association of Nonalcoholic Fatty Liver Disease (NAFLD) and Type 2 Diabetes Mellitus (T2DM) has growing incidence in clinical practice, especially in Western countries [1], since both diseases have high individual prevalence [2] and share the same metabolic risk factors (genetic factors, insulin resistance (IR), dyslipidemia, obesity and lifestyle, etc.) [3]. The global prevalence of NAFLD is estimated to be 25%, using diagnostic tools such as liver ultrasound (US), computer tomography scan (CT), or magnetic resonance spectroscopy (MRS)/imaging (MRI) [2]. Additionally, its high prevalence can be attributed to the lack of strategies and policies to address NAFLD at both the national and global levels [4]. The prevalence of Diabetes Mellitus worldwide is estimated at 10.5% of all adults aged 20–79 years [5], of whom over 90% have T2DM [5].

One of the leading causes of mortality amongst these patients is represented by cardiovascular diseases (CVD), followed by liver-related complications (cirrhosis, carcinoma) [6].

NAFLD is defined conventionally as the presence of hepatic fat accumulation that affects >5% of hepatocytes on histological examination or >5.6% on MRS or MRI [7,8] after excluding other causes of steatosis, especially daily alcohol consumption (>30 g for men; >20 g for women) and chronic infection with hepatitis B or C virus [9]. NAFLD incorporates two histologically and clinically different entities: nonalcoholic fatty liver (NAFL) and nonalcoholic steatohepatitis (NASH). The first one is characterized by simple steatosis, while the second one encompasses beside steatosis, ballooning, and lobular inflammation [7,8]. NASH, as the next step in the natural history of NAFL, is associated with higher mortality from CVD [7].

This review focuses on the bidirectional association between NAFLD and T2DM and their intersection represented by IR. We have analyzed the main data published for this chronic liver disease, its prevalence, and mortality rates among the diabetic population. Briefly, we have pointed out the main pathophysiological mechanisms and the risk stratification algorithm for the development of NAFLD/NASH as well as the tools for evaluation of fibrosis. Finally, we have focused on the current therapeutic options for the treatment of NAFLD associated with T2DM.

## 2. Which Is the Correct Term, “NAFLD” or “MAFLD”?

Recently, the committee of the NAFLD Nomenclature Consensus Meeting High-Level Output agreed that any new nomenclature should be: affirmative, accurate, adaptable, adoptable, applicable, and able to define the contribution of alcohol [10]. Their outcomes will be communicated in September 2022.

The etymology of the word “NAFLD” dates back to 1980 when Jurgen Ludwig named the disease after a series of 20 patients which presented the histological characteristics of alcoholic fatty liver disease with no misuse of alcohol. Since then, this “anti-definition” has not been revised, even though a significant time has passed and numerous advancements have been made in our understanding of the causes and pathogenesis of this disease [11].

Eslam et al. proposed a more practical and clinical-based approach by renaming NAFLD as MAFLD, thus allowing a distinguishable entity separated from the current term “non-alcohol” [12]. In the international expert consensus statement, the diagnosis is based on the presence of steatosis (evaluated via imaging, serum biomarkers, or liver histology) associated with one of the following three criteria: body mass index (BMI) status (overweight/obese), T2DM, or metabolic syndrome [13]. The change of disease definition may affect, in a negative way, the existing data on usage of the biomarkers and therapeutic options [10].

## 3. Prevalence of NAFLD in the T2DM Population

Amiri N. et al. estimate that the early half (54%) of the patients with NAFLD associate T2DM [14]. Another study reported that 85.3% of patients with T2DM had simultaneously fatty liver on US, of which 70% probably have NAFLD, after the exclusion of secondary cause [15]. Other investigators assessed the prevalence of hepatic steatosis on US at 42.6% from a randomly selected cohort of patients with T2DM [16].

At the same time, NAFLD has a high prevalence in T2DM subjects. Thus, based on a meta-analysis published in 2019 that included 80 studies, the estimated global prevalence of NAFLD in patients with T2DM was reported at 55.48% (95%; CI: 47.26–63.67%) [17]. The highest rates were in Europe (68%) and West Asia (67%), while the lowest prevalence was in Africa (30%) [17].

Taken together, in the majority of patients, NAFLD and T2DM coexist and interact, leading to more severe outcomes (i.e., hepatocellular carcinoma [3]; CVD [6]).

Data from a meta-analysis including approximately 300,000 individuals followed over a period of 5 years have shown that individuals with NAFLD had a two-fold greater risk of incident T2DM than those without NAFLD. This risk increases across the higher fat and the fibrosis liver scores [18] and accelerates the development of CVD [15]. On the other hand, the presence of T2DM increases the risk of NASH, cirrhosis, or hepatocellular carcinoma [19].

## 4. Pathophysiological Processes in NAFLD

### 4.1. Genetic Factors

There is a large body of scientific evidence that confirms the genetical component of the fatty liver disease. In this regard, it has been observed that first-degree relatives of patients with NAFLD are at a higher risk for the disease [20]. The most acknowledged genes involved are represented by: Patatin-like phospholipase Domain-Dontaining protein 3 (PNPLA 3), Membrane Bound O-Acyltransferase Domain Containing 7 (MBOAT 7), Glucokinase regulatory protein (GCKR), and Transmembrane 6 superfamily member 2 (TM6SF2), all of which are involved in the hepatic lipid metabolism [21]. Furthermore, the local prevalence of PNPLA 3 genetic variants was associated with the high prevalence of NAFLD in various countries [22] as well as with an increased risk for the development of hepatocellular carcinoma [7].

Also, the fat mass and obesity-associated gene (FTO) were identified to be linked with obesity risk (and probably with NAFLD as well) [23]. Higher expression of Iroquois homeobox (IRX) genes 3 (IRX3) and 5 (IRX5), which are effectors of the FTO variants, induce the transformation of the brown adipocytes (energy-dissipating) into white adipocytes (energy-storing) [24]. Data from animal models have shown that a high-fat diet is associated with an overexpression of FTO in hepatocytes (which promotes the development of NAFLD) [25].

### 4.2. IR

The liver plays a main role in glucose metabolism, making glucose available for other organs in the fasting state. The hepatic production of glucose is regulated by insulin, which influences the processes of glycogen-synthesis/lysis and gluconeogenesis (GNG) [26]. In the postprandial state, insulin secretion spikes as a result of the increase in blood glucose and the incretin hormones, mainly Glucagon Like-Peptide-1 (GLP-1) and Gastric Inhibitory Polypeptide (GIP). Insulin is delivered to the liver through the portal vein, where it suppresses GNG, as a limiting mechanism for the increase in plasma glucose.

Decreased insulin secretion resulting in insulin levels unable to overcome liver IR leads to the loss of inhibition of GNG and glycogenolysis, with an overproduction of glucose. People with obesity have an impairment in the ability of insulin to suppress GNG but often have normal basal and postprandial hepatic glucose production rates because of increased insulin secretion [27,28]. On the other hand, patients with T2DM have a decrease in insulin secretion, leading to high production of hepatic glucose [29].

An indirect effect on hepatic glucose metabolism is caused by the adipose tissue IR and its altered suppression of lipolysis [26]. This increases the release of free fatty acids that are delivered to the liver and represent the substrates for the GNG.

Furthermore, obesity predisposes to a state of low-grade chronic inflammation that mainly takes place in the visceral adipose tissue.

Data suggest that IR represents a consequence of the conversion of adipose tissue macrophages from an M2-like state (anti-inflammatory) to a proinflammatory, M1-like state, presumably due to the increased level of interferon-gamma (INF-γ). This is produced by natural killer cells (NK cells) and by T cells, especially CD8+ [30]. These proinflammatory cells are recruited as a result of the stress produced by obesity-induced adipocyte hypertrophy and hyperplasia, as well as of the shift in adipokine production from adiponectin to leptin and monocyte chemo-attractant protein-1 (MCP-1).

An Interesting link between IR and NAFLD is defined by the presence of certain genes, such as ectoenzyme nucleotide pyrophosphate phosphodiesterase 1 (ENPP1 121 Gln) and the Insulin Receptor Substrate-1 (IRS-1) 972 Arg polymorphism that decreases the activity of protein kinase B (AKT) of the insulin receptor in hepatocytes [31]. This pathophysiological mechanism leads to impaired hepatic insulin signaling, which predisposes to liver damage [31].

In one cross-sectional study, a threshold for the accumulation of intrahepatic triglyceride (IHTG) of approximately 6+/−2% on proton-MRS (1H-MRS), after which metabolic changes such as muscle IR, hypertriglyceridemia, and low high-density lipoprotein cholesterol (HDL-cholesterol) develop, was observed [32]. Likewise, Xia et al. concluded that a liver fat content >20% on US is associated with an increased carotid artery intima-media thickness as a marker of atherosclerosis [33].

### 4.3. Proatherogenic Lipid Profile

As a consequence of the adipose tissue dysfunction, the adiponectin levels decrease, inducing an IR state [34]. Additionally, the reduction of adiponectin levels decreases the free fatty acids (FFA) oxidation, allowing its influx into the liver, as well as stimulating the GNG and de novo lipogenesis [35]. Once in the hepatocyte, the FFA can undergo a beta-oxidation process in the mitochondria or can be esterified to produce triglycerides (TG), then either stored as droplets into the hepatocytes or packaged in apolipoproteins and sent as very low-density lipoproteins (VLDL) in the serum. The excessive oxidation of the FFA produces reactive oxygen species (ROS), together with the proinflammatory state (induced by the IR) and the de novo lipogenesis, which accelerates the formation of atherosclerotic plaque [34].

Patients with NAFLD and T2DM develop a specific, atherogenic lipid profile defined by low levels of HDL-cholesterol as well as elevated concentrations of very low-density lipoproteins, triglyceride, and apolipoprotein B100 (apoB100) [36]. A compensatory mechanism for reducing liver fat content is represented by the overproduction of VLDL particles which serve as transporters of TG to the peripheral tissues [36]. This process stimulates the activity of cholesteryl ester transfer protein (CETP). The main role of this enzyme is to exchange the TG/cholesterol esters between VLDL, HDL-cholesterol, and LDL-cholesterol. Finally, this mechanism results in abnormal HDL-cholesterol metabolism, causing low HDL-cholesterol levels as well as alterations in lipoprotein profile, with an excess of small and dense LDL-cholesterol particles [37].

### 4.4. Gut Microbiota

It seems like Western diets with their high content of fats and sugars such as fructose represent the starting point in the pathogenic process of obesity, T2DM, and NAFLD. They share common gut-microbiome alterations, with a shift toward the Gram-negative bacteria such as Proteobacteria (including *E. coli*), and a decrease in the population of Lactobacillus [37]. Additionally, data have shown that among patients with NAFLD, the Firmicutes/Bacteroidetes ratio (F/B ratio) decreases, as a marker of dysbiosis, and is positively correlated with steatosis [38,39].

Gut dysbiosis determines increasing intestinal permeability which leads to bacterial product translocation such as lipopolysaccharide (LPS), a cell wall component of Gram-negative bacteria, causing the progression of liver fibrosis by activating inflammatory pathways [40,41].

The decrease in the choline bioavailability as well as the presence of its metabolite, trimethylamine-N-oxide (TMAO), are associated with the development of NAFLD by inducing inflammation and liver damage. Choline is an essential nutrient that is metabolized by the gut microbiota to trimethylamine. In turn, trimethylamine is transported to the liver, where it is oxidated to TMAO [42,43].

## 5. Mortality

NAFLD is associated with high all-cause mortality and CVD mortality. Data suggest that 33% of patients with NAFLD died or underwent liver transplantation after a median follow-up of 12 years [44]. Interestingly, Vilar-Gomez E. et al. reported in a small study of 458 patients with biopsy-confirmed advanced fibrosis (stage F3 and F4), followed-up over a mean of 5.5 years, that 50% of deaths of those with bridging fibrosis (F3) were directly attributed to vascular events or nonhepatic cancers [45]. The United States National Health and Nutrition Examination Survey (NHANES) included 11,154 participants, 4083 of whom were NAFLD patients diagnosed by US, with a mean follow-up of 14.5 years. This prospective study concluded that simple steatosis did not increase CVD mortality risk, whereas fatty liver with evidence of advanced fibrosis, evaluated by noninvasive marker panels such as Fibrosis-4 Index (FIB-4), AST to Platelet Ratio Index (APRI), or NAFLD Fibrosis Score (NFS), was associated with increased mortality essentially driven by CV causes [46].

The presence of T2DM or an advanced form of fibrosis (>F3) adds an extra CVD mortality risk [36].

## 6. Diagnosis

It is unanimously agreed by the different guidelines that patients with T2DM are at increased risk for NAFLD, NASH, and advanced fibrosis [7,8]. In this regard, the 2016 European Association for the Study of the Liver (EASL) guideline suggested screening using liver enzymes and/or US in all patients with obesity or metabolic syndrome and especially those with T2DM [7]. By contrast, the American Association for the Study of Liver Diseases (AASLD) guidelines proposed a more targeted screening that includes only the cases “with a high index of suspicion” for the development of NAFLD or NASH [8]. This recommendation is due to the uncertainties regarding the diagnosis, natural history, and treatment. Additionally, the AASLD suggested the usage of noninvasive tests such as NFS, FIB-4, or vibration controlled transient elastography (VCTE) for the stratification of fibrosis risk [8].

Recent data indicate that screening and risk-stratification pathways for NAFLD are cost-effective among patients with T2DM [47]. Considering the respective data, the American Gastroenterological Association (AGA) proposes screening for NAFLD and risk stratification for advanced fibrosis and also in lean patients with T2DM [48]. NASH represents the next stage in the natural evolution of NAFLD, involving up to 20% of these patients [49]. Histologically, it is defined as more than 5% hepatic steatosis as well as evidence of hepatocyte damage (ballooning) and lobular inflammation, with or without fibrosis [8].

According to the AASLD, liver biopsy is recommended in patients with NAFLD at a high risk (metabolic syndrome, T2DM) of having NASH and/or hepatic fibrosis, particularly in cases where a chronic liver disease coexists. Despite the cost, sampling variability, and procedure-related morbidity (bleeding, infections, bile leak) and mortality (<0.01%) [50], liver biopsy remains the gold standard for the diagnosis of NASH [7,8].

Given the increased prevalence (38%) in T2DM patients, the American Diabetes Association (ADA) 2022 recommends screening for NASH and liver fibrosis in all patients with T2DM or prediabetes and elevated liver enzymes or fatty liver on US [17].

The NAFLD Activity Scoring (NAS) system was proposed and validated in 2005 by the NASH Clinical Research Network Pathology Committee as a tool for evaluating the disease severity, rather than for the diagnosis of NASH [51]. This only includes features of active injury (lobular inflammation and ballooning) that are potentially reversible [51].

Another score used to assess the grade of steatosis, activity, and the stage of fibrosis is the Steatosis, Activity, and Fibrosis (SAF) score. One of its main advantages is that it describes the above three histopathological features in a practical manner, being able to classify some special subgroups (i.e., patients with steatosis and fibrosis and no cell injury) [52]. Additionally, the SAF score is beneficial in clinical trials where the changes observed in paired biopsies are easily quantified [8,52]. As a conclusion, no scoring system can comprehend all the information provided by the liver biopsy.

## 7. Evaluation of Fibrosis

Long-term liver-related outcomes are associated with the degree of hepatic fibrosis [44]. Stratification of the risk for advanced fibrosis in a population with numerous metabolic cofactors can be assessed in the primary care clinics using a two-step approach [53]. The main two tests recommended are the FIB-4 score and the transient elastography (TE) [53].

Firstly, the usage of the FIB-4 with a cut-off value of more than 1.30 identifies the patients with intermediate or high risk who would benefit from the TE. On the other hand, patients with a FIB-4 score of less than 1.30 should receive recommendations for lifestyle optimization and retesting in 1–3 years [53].

The FIB-4 is widely available and easily used because it is based on common clinical parameters (age) and biological parameters (transaminases and platelet count). Furthermore, the FIB-4 index with a cut-off of 1.30 has been reported as having a high negative predictive value (≥80%) for advanced fibrosis regardless of the presence of T2DM [54].

The second assessment, using the TE technique, is a physical approach based on the measurement of liver stiffness. At a value lower than 8 kPa, the patient is considered at a low risk for advanced fibrosis, while any value higher than 8 kPa places the patient in the at-risk category. A high value must be confirmed by using a patented serum test such as Enhanced Liver Fibrosis (ELF), FibroMeter, or Fibrotest 0.48, unless the only recommendation is to consider liver biopsy. In case of discordance between the serum test and the TE test, then a liver biopsy can be taken into account. Patients with confirmed liver stiffness are likely in stages F3–F4 of advanced fibrosis [53].

## 8. Management

### 8.1. Nonpharmacological Interventions

A growing body of evidence supports that weight loss is the major therapeutic target in NAFLD [55].

A reduction of 3–5% of body weight improves hepatic steatosis [8] and reverses hepatic IR [56], whereas a weight loss of 7–10% can lead to significant rates of resolution of NASH due to the amelioration of hepatic inflammation [57]. These data are supported by a 12-month prospective trial that was based on paired liver biopsies [57]. Additionally, this dose-response relationship has shown that a >10% reduction of the body weight was associated with fibrosis regression [36,57]. Unfortunately, 70% of the patients included in the abovementioned trial lost less than 5% of weight, mostly as a result of poor adherence and compliance to weight loss recommendations.

Despite the minimal or no weight loss, physical exercise intervention in NAFLD patients has shown to reduce hepatic steatosis [58], probably due to reducing the lipogenic enzymes acetyl-Co-enzyme A carboxylase and fatty acid synthase, as seen in rodent studies [59]. Regarding the duration and intensity of the exercise program, further data are required in order to standardize this recommendation. Considering the fact that all exercise trials are small, with low statistical power, it has been observed that neither form of exercise (aerobic exercise, resistance exercise, or high-intensity intermittent exercise) has shown additional benefits in NAFLD management. Data from a randomized clinical trial confirm that the IHTG has been reduced slightly, by 5.0% (95%; CI: −7.2% to 2.8%; *p* < 0.001) in the vigorous-moderate exercise group and 4.2% (95%; CI: −6.3% to −2.0%; *p* < 0.001) the moderate exercise group versus the control group at the 6 months assessment [60]. Additionally, it has been observed that physical activity with a duration (≥150 min/week) was associated with a 44% lower risk of developing NAFLD (OR: 0.56; 95%; CI: 0.46–0.67; *p* = 0.001). Those patients who reported 1–2 times as much (150–299 min/week) or over 2 times as much (≥300 min/week) had 40% (OR: 0.60; 95%; CI: 0.41–0.90; *p* = 0.016) and 49% (OR:0.51; 95%; CI: 0.40–0.65; *p* < 0.001) of developing NAFLD, respectively [61].

However, there is evidence that patients who are physically active for more than 150 min/week or who increase their activity level by more than 60 min/week over a period of 3 months have an improvement in liver enzymes independent of the weight loss [62]. In addition, a sustainable exercise program has proven benefits on the main risk factors associated with fatty liver such as T2DM [63] and visceral adiposity [64].

As mentioned above, being overweight and obesity are major contributors and risk factors for the development of NAFLD; therefore, calorie-restricted diets have been considered as possible nonpharmacological tools for NAFLD treatment. Data suggest that a reduction of 500–1000 kcal/day, regardless of the macronutrient composition, leads to decreases in IHTG content and hepatic IR in the first 48 h along with suppression of endogenous glucose production [7,65]. In the long-term (11 weeks), it increases muscle insulin-mediated glucose uptake [65].

Increasing evidence supports the benefits of the Mediterranean diet. This diet includes plenty of vegetables, legumes, and fruits along with whole grains and nuts. It encourages switching from red meat and processed meat to fish and seafood. The Mediterranean diet also has numerous benefits due to its anti-inflammatory and antioxidant components such as polyphenols, which are present in whole-grain cereals, vegetables, fresh fruits, olive oil, nuts, and vitamins [66].

A useful tool in assessing the proinflammatory or anti-inflammatory properties of the food is the dietary inflammatory index [67]. The DII score ranges from 7.98 (maximal proinflammatory dietary) to −8.87 (maximal anti-inflammatory dietary pattern) [67]. Regarding types of diet, the macrobiotic and the Mediterranean diets have low DII scores (−5.54 and −3.98, respectively). These elements could explain the benefits of these diets [67].

Evidence from a small randomized trial that included biopsy-proven NAFLD patients demonstrated a decrease in the hepatic steatosis (assessed by MRS) as well as improvement in insulin sensitivity (IS) [68]. More data from a meta-analysis confirm this beneficial role of the Mediterranean diet in terms of decreasing the FLI (−1.06; 95% CI: −1.95 to −0.17; *p* = 0.02) and IR (−0.34; 95% CI: −0.65 to −0.03; *p* = 0.03) compared with the control group [69]. Additionally, it is acknowledged that following the Mediterranean diet can improve the glycemic control and CV risk in patients with established diabetes [70].

### 8.2. Vitamin Supplements

In animal models, vitamin D enhances the intracellular mechanisms of insulin action mediated by IRS-1 and vitamin D receptors, facilitating the glucose uptake in muscle and promoting the expression of insulin-dependent glucose transporter 4 (GLUT 4) on adipocytes [71]. Additionally, at the hepatic level, vitamin D inhibits the proliferation of hepatic stellate cells and the expression of profibrotic mediators such as the platelet-derived growth factor (PDGF) and the transforming growth factor β (TGF-β). These findings indicate vitamin D antifibrotic action [72].

Although epidemiological studies confirm the association between reduced levels of 25-OH vitamin D and the presence of NAFLD [73,74], there are no clinical data from large population in support of its benefit. Small studies such as the study of Barchetta I. et al., which included 65 patients with T2DM and NAFLD, could not validate any improvement of the hepatic steatosis or metabolic/cardiovascular parameters following the usage of high-dosage vitamin D supplements (2000 UI/day) [75].

Current guidelines [7,8] do not recommend vitamin E as a treatment option for patients with T2DM with NASH because of the lack of supporting data, as well as because of the concerns about long-term safety due to its high risk of hemorrhagic stroke [76] and prostate cancer [77]. In addition, a small randomized controlled trial that included 105 patients with biopsy-proven NASH and T2DM has shown no histological improvement after the administration of vitamin E (400 IU/day) for 18 months [78].

### 8.3. Pharmacological Therapy

Due to the effect of IR on hepatic and adipose tissue, pharmacological therapies that improve IS are an option for patients who associate NAFLD and T2DM. In addition, weight loss induced by some diabetes drugs is expected to induce improvements of NAFLD in T2DM.

Generally, the first pharmacological line therapy for patients with T2DM is represented by metformin [79]. Its benefits on IR, by decreasing the rate of GNG and stimulating the muscular glucose uptake [80], made it a potential treatment for NAFLD. Data from a systematic review that included patients with NAFLD have shown that metformin improves the biochemical panel by reducing the liver enzymes and decreasing IR and BMI [81]. In patients with NAFLD and T2DM, metformin associated with lifestyle intervention promotes weight loss with a mean of 4.3–7.9% of initial body weight, leading to amelioration of hepatic IS, and improves glycemic control as well [82]. Unfortunately, metformin showed no efficacy on NAFLD’s histological aspect [81,83].

Thiazolidinediones increase IS and are a potential treatment option for NAFLD. Pioglitazone acts as an agonist of the peroxisome proliferator-activated receptor gamma (PPARγ), which are abundant in the adipocytes and rare in muscle [84]. The activation of these nuclear receptors results in stimulation of the adipogenesis by promoting TG storage, improves gene transcription of key enzymes involved in lipogenesis, and enhances the suppressive action of insulin on lipolysis [84,85].

Data have confirmed that the administration of pioglitazone in patients with T2DM and NAFLD increases the plasma adiponectin concentration [86], leading to a decrease in IHTG and improvement in glycemic control [87]. Additionally, regarding NASH, data have shown that thiazolidinedione therapy was associated with amelioration of advanced fibrosis (OR: 3.15; CI: 1.25–7.93; *p* = 0.01), fibrosis of any stage (OR: 1.66; CI: 1.12–2.47; *p* = 0.01), and resolution of NASH (OR: 3.22; CI: 2.17–4.79; *p* < 0.001). Although the benefit of thiazolidinediones on NAFLD and NASH is well-established, their usage is limited now mainly because of their side effects, especially weight gain, lower limb edema, and high risk for bone fracture [88].

GLP-1 is an incretin hormone secreted from the gut that stimulates the production and release of insulin, inhibits glucagon secretion (both glucose dependent), delays gastric emptying, and reduces appetite and food intake [89]. Its half-time life is short due to the effect of the dipeptidyl peptidase 4 (DPP4) enzyme that cleaves the GLP-1 molecule.

Given their glucose lowering and cardioprotective effect, GLP-1 receptor agonists (GLP-1 RAs) can represent additional or alternative first-line therapeutic agents for patients with T2DM and established atherosclerotic cardiovascular disease (ASCVD) or at high risk of CV/renal complications [79].

Clinical data suggested that exenatide added to the antidiabetic treatment decreased IHTG significantly, with 23.8 ± 9.5% in correlation with weight loss (r = 0.47, *p* = 0.03) [90].

Data from the phase IIb Liraglutide Efficacy and Action in NASH (LEAN) study have demonstrated that liraglutide proved benefits in the resolution of NASH without any worsening of fibrosis (39%) versus placebo (9%) (RR: 4.3; 95% CI: 1.0–17) [88]. Additionally, 83% of patients on liraglutide 1.8 mg daily, for 48 weeks, improved steatosis versus placebo (45%; *p* = 0.009), and 61% improved hepatocyte ballooning versus 32% (*p* = 0.05) [91]. These effects are supposed to be mostly due to the fact that liraglutide reduces hepatic de novo lipogenesis in vivo, a major component in the pathogenesis of NAFLD [92].

Semaglutide is approved for the treatment of T2DM and has shown benefits in the management of patients with obesity/overweight [93]. Additionally, data from a double-blind, placebo controlled, phase 2 trial, which included patients with biopsy confirmed NASH and liver fibrosis stage F1 to F3, has revealed that 0.4 mg of semaglutide weekly was associated in 59% of cases with a resolution of NASH versus 17% in the placebo group (CI: 2.60–17.63; *p* < 0.001) [94].

Likewise, data from D-LIFT (Effect of Dulaglutide on Liver Fat), a small study that assessed the effect of dulaglutide on liver fat content, showed that dulaglutide achieved a reduction of −3.5% (95%; CI: 6.6−0.4%; *p* = 0.025) among patients with T2DM and NAFLD, corresponding to a 2.6-fold greater decrease versus controls [95].

Data seem to indicate that improvement in NAFLD can be in part attributed to the GLP-1 RAs’ effect on body weight loss [96].

Encouraging data come from the novel GIP and GLP1-RAs, tirzepatide. Higher tirzepatide doses (15 mg) showed significantly decreased levels of NASH-related biomarkers such as K-18 and Pro C-13 [97].

Sodium-glucose cotransporter 2 (SGLT 2) inhibitors are oral antidiabetic drugs that promote urinary excretion of glucose by inhibiting its renal proximal tubular reabsorption.

The reduction of the risk of major cardiovascular events (myocardial infarction, stroke, cardiovascular death) for those patients with ASCVD, as well as the reduction in hospitalization for heart failure and delayed chronic kidney disease (CKD) progression, makes SGLT 2 inhibitors a potential first line treatment for patients with T2DM [98].

Patients with NAFLD and T2DM treated with either canagliflozin, dapagliflozin, or empagliflozin showed a decrease in HBA1c value and a decrease in the IHTG and in some liver enzymes [99,100,101]. Additionally, dapagliflozin proved benefits versus placebo in improving liver steatosis and fibrosis evaluated using TE among a small cohort of 63 patients with T2DM and NAFLD [102].

Due to the fact that neither GLP-1 RAs nor SGLT 2 inhibitors are expressed in the liver, the improvement of the hepatic lipid content is mainly a consequence of weight loss [103]. The association of GLP-1RAs with SGLT2i is expected to improve the benefits on NAFLD [104].

### 8.4. Surgical Treatments for Obesity

For patients with obesity and/or T2DM and NAFLD that had no liver improvement after lifestyle intervention and pharmacological treatment, surgical procedures for obesity management remain a procedure of last resort. According to the ADA 2022 guidelines, metabolic surgery is recommended to treat T2DM in patients with class III obesity (BMI ≥ 40 kg/m^2^) and can also be considered as an option for those with a BMI between 30–39.9 kg/m^2^ who do not reach glycemic targets, achieve long standing weight loss, and improve their comorbidities [105].

Clinical data from patients with obesity and hepatic steatosis suggest that one year after bariatric surgery, the metabolic syndrome and hepatic steatosis were improved [106]. The median decrease in steatosis was 8.5% (CI: 4–10; *p* < 0.0001), probably due to the significant weight loss associated with bariatric surgery [106]. Furthermore, it assessed the connection between IR and the severity of preoperative steatosis as well as its improvement after surgery [106]. This observation can lead to a better identification of patients with obesity that can benefit from metabolic surgery [106].

Regarding the type of surgical procedures, clinical data demonstrated that the NAS score was substantially decreased at 1 year and 5 years after the surgery, especially in those who underwent gastric bypass (Roux-en-Y gastric bypass) versus adjustable gastric banding [107].

Endoscopic Bariatric and Metabolic Therapies (EBMTs) represent less invasive methods that aim to achieve weight loss with results comparable to bariatric surgery, at a more affordable cost and with a lower risk of complications. The most well-known techniques are represented by: inserting an intragastric balloon (IGB), endoscopic sleeve gastroplasty (ESG), and aspiration techniques (AT). Khan et al. confirm in their meta-analysis the utility of these new treatment options. It was observed that the pooled mean of the total body weight loss, at 12 months, for the ESG was 17.41 (95%; CI: 17.08–17.74; $I^{2}=0\%$), and for the AT was 15.37 (95%; CI: 9.00–21.74; $I^{2}=80\%$) [108].

Regarding their efficacy on the NAFLD, IGB was associated with an improvement of the NAS (MD–3 (95%; CI: −3.27 to −2.73) [109].

## 9. Conclusions

Globally, NAFLD affects almost a quarter of the population, though its prevalence may vary with the used diagnostic tool. Despite its high prevalence, frequent association with T2DM, and high mortality rates (mostly due to CV causes), NAFLD remains underdiagnosed and underevaluated, mainly due to the lack of specific public health strategies.

The prevalence of NAFLD in T2DM individuals is higher than in the general population. NAFLD doubles the risk of incident T2DM, varying with the fat and fibrosis liver scores, and accelerates the development of CVD. On the other hand, the presence of T2DM increases the risk of fatty liver progression to NASH, cirrhosis, or hepatocellular carcinoma [17].

Given the risk of NAFLD progression in patients with T2DM, international societies have proposed algorithms based on the evaluation of hepatic fat using US and the levels of liver enzymes and stratifying those according to the risk. Therefore, in patients with abnormal liver enzymes and high fat liver content, additional fibrosis evaluation must be taken into consideration, due to the increased CV risk.

Regarding the treatment options for the patients that associate both NAFLD and T2DM, lifestyle intervention and weight loss represent the cornerstone, ameliorating not only the steatosis and fibrosis stage, but also improving glycemic control and leading to metabolic health. Many current antidiabetic drugs that target IR or bring benefits of weight loss have yielded positive results in the management of NAFLD patients.

Overall, in the near future, more clinical data is needed in order to make a firm recommendation about the optimal treatment for the patients that associate NAFLD and T2DM.

## Data Availability

Not applicable.
